# Effectiveness of Interoceptive Programs to Improve Academic Self-Regulation and Reduce Behavioral Problems Among Children With Learning Disabilities

**DOI:** 10.7759/cureus.61816

**Published:** 2024-06-06

**Authors:** M Arun Kumar, Swathi G

**Affiliations:** 1 Occupational Therapy, Saveetha College of Occupational Therapy, Saveetha Institute of Medical and Technical Sciences, Saveetha University, Chennai, IND

**Keywords:** behavioral problems, self regulation, interoceptive activities, interoception, specific learning disability, learning disability

## Abstract

Background

Learning disabilities occur in very young children, yet they are usually not noticed until the child reaches school age. These issues can affect the child’s ability to perform activities of daily living and their ability to learn including academic activities. Occupational therapy can assist with improving their ability to learn which will also help their performance at school.

Aims and objectives

The present study was designed to investigate the impact of interoceptive programs on improving academic self-regulation and reducing behavioral problems among children with learning disabilities. The objectives of the study were to determine a learning disability through the use of the Learning Disability Checklist. The Academic Self-Regulation Questionnaire for Learning Disability (SRQ-A LD) was used to assess the degree of academic self-regulation, while the Behavior Rating Inventory for Executive Functioning-2 (BRIEF-2) was used to assess the degree of behavioral problems. The primary objective was to evaluate the efficacy of interoceptive programs to enhance academic self-regulation and lessen behavioral issues in the experimental group, as well as to find out the impact of conventional occupational therapy for children with learning disabilities in the control group.

Methodology

This was a quasi-experimental design with convenience sampling and selected samples (n=50), which were then split into two groups, an experimental group (n=25) and a control group (n=25), based on the inclusion and exclusion criteria. The experimental group received intervention based on interoception activities, while the control group received conventional occupational therapy. A pre-test and post-test were conducted to measure the changes. The study assessed 50 children in the range of 7 to 11 years, using an initial Madras Dyslexia Association Checklist. Academic self-regulation was assessed using SRQ-A LD. Behaviors were assessed using BRIEF-2. The duration of the study was six months, three sessions per week, lasting 45 minutes to an hour each. The statistical analysis was done with significance at a 1% alpha level using IBM SPSS version 29.0 (Armonk, NY: IBM Corp).

Results

The pre-test and post-test data were analyzed using the Wilcoxon signed-rank test and the Mann-Whitney test. The study found that the interoceptive program had an impact on academic self-regulation and behavioral problems among children with learning disabilities. The p-value of academic self-regulation was <0.001, and the p-value of the behavior rating inventory was <0.001, the alternate hypothesis was accepted. Thus, the results showed significant improvement in academic self-regulation and behavioral problems in the experimental group compared to the control group.

Conclusion

The results of the study showed that the Interoceptive program helped children with learning disabilities improve their academic self-regulation and have fewer behavioral issues. Interoception can positively regulate their behavior. For this reason, the primary therapeutic approach for children with learning disabilities can be the implementation of Interoceptive activities.

## Introduction

Occupational therapy is a client-centered health profession concerned with promoting health and well-being through occupation. The primary goal of occupational therapy is to enable people to participate in the activities of everyday life. Occupational therapists achieve this outcome by working with people and communities to enhance their ability to engage in the occupations they want to, need to, or are expected to do, or by modifying the occupation or the environment to better support occupational engagement for those who are restricted in their participation or who are socially excluded owing to their membership in social or cultural minority groups [1]. Occupational therapists believe that participation can be supported or restricted by the physical, affective, or cognitive abilities of the individual, the characteristics of the occupation, or the physical, social, cultural, attitudinal, and legislative environments. Therefore, occupational therapy practice is focused on enabling individuals to change aspects of their person, the occupation, the environment, or some combination of these to enhance occupational participation. Occupational therapy, often referred to as OT, is an integral part of the healthcare system [2]. Children who receive occupational therapy are encouraged to participate and engage in their daily roles. Children's responsibilities include play and leisure activities, becoming productive, and gaining personal independence. Social isolation might result from being unable to engage due to illness, a disability, or a lack of skills and a diminished sense of self. When choosing interventions for children, occupational therapists consider how the children function in everyday activities, how their disability affects that performance, and how their surroundings either enhance or limit that performance [3].

The term learning disabilities describes a group of problems that affect the ability of a child to master school tasks, process information, and communicate effectively. These disabilities are often not associated with a specific neurologic insult and may or may not be accompanied by an intellectual disability. Learning disabilities are associated with a variety of other neurologic problems, for example, attention deficit disorder and attention deficit disorder with hyperactivity. Specific learning disabilities include auditory processing, language disabilities, and perceptual impairments [4]. The definition of learning disabilities has changed over the years, the focus of attention has shifted from the medical and psychiatric areas to education and psychology. According to the following definition taken from Public Law, learning disability is one of the more basic psychological processes involved in understanding or using language, spoken or written, which may manifest itself in an imperfect ability to listen, think, speak, read, write, or do mathematical calculations [5]. In children, learning problems affect one or more out of every 59. When compared to children without disabilities, children with learning difficulties are 31% more likely to be bullied. Roughly 66% of kids with learning disabilities are diagnosed as boys. More than 18,000 students with learning disabilities leave school early [6]. Early identification can increase the likelihood that the child will benefit from therapy or treatment. Although a learning disability cannot be cured, children can succeed in school and in life if they receive timely assistance and intervention. When a child struggles with managing schoolwork and life skills, parents and teachers are usually the first to notice. Occupational therapists are passionate about their unique role in working with people with learning disabilities. Occupational therapy training uniquely covers both health and social care, with an emphasis on both physical and mental health [7,8]. Students in regular and special education programs may get help from occupational therapists working in public school settings.

Occupational therapy professionals can utilize sensory-based interventions or a sensory integration approach to support a child's participation in their educational program when the child's ability to process and integrate sensory information impedes their performance in school activities [9]. One of the internal senses is interoception, which refers to the ability to take in information about the inside state of the body from the skin and organs. Researchers have discovered important insights into the ways in which interoception interacts with social skills, self-regulation, emotional identification, emotional control, and nearly all everyday tasks, despite the fact that it is a highly developed and complex kind of sensory perception. Interoception is a sensory-motor capacity that is currently receiving more attention in occupational therapy studies. Many academics are investigating the role of interoception in everyday actions. Additionally, researchers that investigate interoception-based skills are presently developing or constructing assessments for skills connected to sensory processing. Higher degrees of cortical processing might not be necessary for interoceptive information. In other situations, interoceptive signals have the potential to initiate higher-order cognitive, emotional, and/or perceptual processing, which can lead to the conscious awareness level [10,11]. This field includes the interoception program, which aims to cultivate an interoceptive practical application, Those whose interoception is still developing may find it difficult to identify the physiological signs that signal mood swings or the necessity of physical self-regulation [12]. One mindfulness-based technique called interoception has been shown to stop episodes of dysregulation and stop impulsive behavior. Studies show that practicing mindfulness reduces emotional distraction and cognitive inflexibility, increases emotional and cognitive awareness, and facilitates the deliberate control of behavior, attention, and emotion [13]. Fundamental to mindfulness, interoception may be the main way that practitioners experience the benefits of the practice. An immediate experience of body sensations replaces thinking about body sensations, and this is thought to be the outcome of most mindfulness training and mindfulness-based interventions. This is because they promote interoception awareness. The growth of the insula and associated neural networks may nurture dispositional mindfulness. Mindfulness and some of its advantages may be better understood as heightened interoception as a result of the neuroplasticity changes in the insula. There is a limited body of literature supporting the term interoception. Interoception is a newly-developed term, which needs more research to enhance the learning process of children [14,15].

## Materials and methods

The study received ethical approval from the Institutional Scientific Review Board of Saveetha College of Occupational Therapy (SCOT/ISRB/002/2023). This research is a quantitative study with a quasi-experimental design. This quasi-experimental study included an experimental group and a control group. This research was conducted in the district of Kanchipuram, Tamil Nadu, India. Children from various clinics, which include the Pupil Saveetha Eco School, Montfort Matriculation School, Sherin’s Rainbow Center, and Senthil Occupational Therapy Center of this district, took part in this study. The sampling technique used was non-randomized convenience sampling. Convenience sampling is useful for obtaining samples and selecting sample representatives from all elements when the population has a wide distribution. The sampling is based on a predetermined population area. The sample size was calculated using the formula n = z2 * p * (1 - p) / e2; where: z = 1.28 for a confidence level (α) of 80%; 5% was chosen as the margin of error. With an estimated 8% incidence (population proportion) of specific learning disorders (SLD) in India, the study required a sample size of 49 [16]. The attrition rate for learning disabilities was found to be 2.04%. Therefore, a sample size of n = 50, in the range of seven to 11 years, was determined. Samples were selected based on the inclusion and exclusion criteria. Then they were divided into 25 participants in the experimental group and 25 participants in the control group. Samples were to be collected according to the criteria. Subjects for the study included both male and female participants between the ages of 7 and 11 who were regular attendees at the schools and who met the criteria listed in the Learning Disability Checklist and Diagnostic and Statistical Manual-5. The study excluded subjects who had less than seven years of experience. Children involved in any extra coaching classes, like tuition, and subjects having difficulty understanding and following commands were excluded. The data collection methods used in this research included tests, questionnaires, and documentation. Various assessments were used to evaluate their learning abilities, academic self-regulation, and behavioral problems. These tests were administered as pre- and post-tests. Samples were selected according to the criteria of the Madras Dyslexia Association Checklist and Diagnostic and Statistical Manual-5. Academic self-regulation was assessed using the Academic Self-Regulation Questionnaire for Learning Disabilities (SRQ-A LD), with a validity of 0.88 and a reliability of 0.96. Behavioral problems were assessed using the Behavior Rating Inventory for Executive Functioning-2 (BRIEF-2), with a validity of 0.98 and a reliability of 0.80 [17,18]. An interoception program and activities involving adaptations to the muscular system, including respiration, temperature, pulse, and touch, were administered to the experimental group. They were encouraged to complete summative, comprehension, and rubric summative activities during the exercise, which assessed their ability to learn, their body's reactions, their knowledge and states of emotions, their self-explanation, and their self-questioning. At the conclusion of each session, as well as the following one, each task was reviewed and explained. Concurrently, the control group received traditional occupational therapy, emphasizing cognitive methods that took into account their learning challenges. Permission to participate in this research was obtained. Twenty-five of the 50 individuals were placed in the experimental group, while the other 25 were placed in the control group. Each group completed a pre-test. Three sessions of treatment were planned over the six-month research period. Both the control and experimental groups completed a post-test at the conclusion of the six-month period. Following that, a statistical analysis was performed on the test findings. The statistical analysis was performed using IBM SPSS version 29.0 (Armonk, NY: IBM Corp.). Both the Mann-Whitney U test and the Wilcoxon signed-rank test were applied. Each scale's pre- and post-test results are compared, and the significance was set at a p-value of 0.01.

## Results

Statistical analysis of pre-test and post-test of SRQ-A LD in the control group

In Table 1, the Wilcoxon Signed Rank Test was performed at a 1% (p < 0.01) significant level. Since the p-value (0.001) was less than 0.01, an alternate hypothesis was accepted. Hence, there was a statistically significant difference in the pre-test and post-test of the SRQ-A LD control group.

**Table 1:** Statistical analysis of pre-test and post-test of SRQ-A LD in the control group SRQ-A LD: Academic Self-Regulation Questionnaire for Learning Disability * p-value indicates significance at the 1% level

Scales		Mean	N	Z value	p-value
SRQ-A LD control group	Pre-test	14.4	25	-3.234	0.001*
	Post-test	15.04	25		

Statistical analysis of pre-test and post-test of SRQ-A LD in the experimental group

In Table 2, the Wilcoxon Signed Rank Test was performed at a 1% (p < 0.01) significant level. Since the p-value (<0.001) was less than 0.01, an alternate hypothesis was accepted. Hence, there was a statistically significant difference in the pre-test and post-test of the SRQ-A LD experimental group.

**Table 2:** Statistical analysis of pre-test and post-test of SRQ-A LD in the experimental group SRQ-A LD: Academic Self-Regulation Questionnaire for Learning Disability * p-value indicates significance at the 1% level

Scales		Mean	N	Z value	p-value
SRQ-A LD experimental group	Pre-test	13.88	25	-4.389	<0.001*
	Post-test	19.12	25		

Mean comparison of SRQ-A LD between the post-test scores of experimental and control groups

In Table 3, Mann-Whitney U was performed at a 1% (p < 0.01) significant level. Since the p-value (<0.001) was less than 0.01, an alternate hypothesis was accepted. Hence, there was a statistically significant difference in post-test scores between the experimental and control groups of the total score.

**Table 3:** Mean comparison of SRQ-A LD between the post-test scores of experimental and control groups SRQ-A LD: Academic Self-Regulation Questionnaire for Learning Disability * p-value indicates significance at the 1% level

Scales		Mean	N	U value	p-value
SRQ- A LD post-test	Control	14.74	25	-5.259	<0.001*
	Experimental	36.26	25		

Statistical analysis of pre-test and post-test of BRIEF-2 in the control group

In Table 4, the Wilcoxon Signed Rank Test was performed at a 1% (p < 0.01) significant level. Since the p-value (<0.001) was less than 0.01, an alternate hypothesis was accepted. Hence, there was a statistically significant difference in the pre-test and post-test of the BRIEF-2 control group.

**Table 4:** Statistical analysis of pre-test and post-test of BRIEF-2 in the control group BRIEF-2: Behavior Rating Inventory for Executive Functioning-2 * p-value indicates significance at the 1% level

Scales		Mean	N	Z value	p-value
BRIEF-2	Pre-test	63.6	25	-3.944	<0.001*
	Post-test	62.52	25		

Statistical analysis of pre-test and post-test of BRIEF-2 in the experimental group

In Table 5, the Wilcoxon Signed Rank Test was performed at a 1% (p < 0.01) significant level. Since the p-value (<0.001) was less than 0.01, an alternate hypothesis was accepted. Hence, there was a statistically significant difference in the pre-test and post-test of the BRIEF-2 experimental group.

**Table 5:** Statistical analysis of pre-test and post-test of BRIEF-2 in the experimental group BRIEF-2: Behavior Rating Inventory for Executive Functioning-2 * p-value indicates significance at the 1% level

Scales		Mean	N	Z value	p-value
BRIEF-2 experimental group	Pre-test	64.76	25	-4.398	<0.001*
	Post-test	58.32	25		

Mean comparison of BRIEF-2 between the post-test scores of experimental and control groups

In Table 6, Mann Whitney U was performed at a 1% (p < 0.01) significant level. Since the p-value of (<0.001) was less than 0.01, an alternate hypothesis was accepted. Hence, there was a statistically significant difference in post-test scores between the experimental and control groups of the total score.

**Table 6:** Mean comparison of BRIEF-2 between the post-test scores of experimental and control groups BRIEF-2: Behavior Rating Inventory for Executive Functioning-2 * p-value indicates significance at the 1% level

Scales		Mean	N	U value	p-value
BRIEF-2 post-test	Control	31.98	25	-3.131	<0.001*
	Experimental	19.08	25		

## Discussion

The purpose of this study was to determine the effect of interoception activities to improve academic self-regulation and reduce behavioral problems among children with learning disabilities. Data from each scale's pre-tests and post-tests were compared. Furthermore, the significance was established at p<0.01. Since the p-value was <0.001 (significant at 1%), an alternate hypothesis was accepted. Hence, there was a statistically significant difference in the pre-test and post-test of the experimental group substantially surpassing the control group in terms of academic self-regulation and behavioral problems. This suggests that engaging in interoception-based activities may be a more effective way to improve academic self-regulation and reduce behavioral problems among children than undergoing traditional occupational therapy education sessions.

Table 1 shows the results of the statistical analysis of the pre-tests and post-tests of the control group on academic self-regulation. Since the p-value was 0.001 (Significant at 1%), in the SRQ-A LD for the control group, an alternate hypothesis was accepted. Hence, there was a statistically significant difference between the pre-test and post-test scores of SRQ-A LD in the control group. This suggests that the children who received conventional occupational therapy in the control group had significant improvement. Previous research implicated the same result, indicating that traditional occupational therapy training improves academic performance [19]. Another study, by Arfé et al., concluded the same way, with children who received occupational therapy training showing significant improvements in reading, spelling, inhibitory control, and executive functioning [20,21].

The findings of the statistical analysis of the experimental group's pre-test and post-test results on academic self-regulation are displayed in Table 2. The alternate hypothesis was accepted since the p-value for the experimental group's academic self-regulation questionnaire was <0.001 (significant at 1%). As a result, the experimental group's pre-test and post-test results on the SRQ-A LD differed significantly in statistics. This implied that there was a notable enhancement in the intervention that the children in the experimental group received. The study implied the same results with the implementation of multisensory techniques: interventions had an overall treatment effect for participants with typical development and dyslexia. So it is essential to incorporate this learning style into the instruction [22,23]. Another study was conducted on interoceptive awareness and self-regulation strategies to enhance self-control and learning engagement, which implied the same results. Self-regulatory interventions have demonstrated numerous benefits for improving the academic performance of students [24,25]. The outcomes of the statistical analysis of the control and experimental groups' post-scores on academic self-regulation are displayed in Table 3. An alternative hypothesis was accepted because the SRQ-A LD's p-value was <0.001 (significant at 1%). Therefore, on the SRQ-A LD, there was a statistically significant difference in the post-test scores between the experimental and control groups. These results were supported by various other studies, which implied that encouraging all children to learn interoceptive activities can have a favorable impact on their self-regulation, academic performance, and engagement [26].

The findings of the statistical analysis of the control group's pre-test and post-tests on the BRIEF-2 scale are displayed in Table 4. The alternate hypothesis was accepted since the p-value for the executive functioning scale on the BRIEF-2 in the control group was <0.001 (significant at 1%). Therefore, a statistically significant difference existed between the behavior scale's pre-test and post-test scores. This implied that the children in the control group who got traditional occupational therapy made considerable progress after receiving the intervention. Additionally, the findings of this study were also supported by a study that aimed to determine the effect of conventional occupational therapy on reducing behavioral problems among children with learning disabilities [27,28]. The findings of the statistical analysis of the experimental group's pre-tests and post-tests on the BRIEF-2 are shown in Table 5. As the experimental group's p-value on the BRIEF-2 was <0.001 (significant at 1%), the alternative hypothesis was approved. Accordingly, the experimental group's pre-test and post-test behavior scale scores differed in statistics significantly. This implied that there was a notable enhancement in the intervention that the children in the experimental group received. Accordingly, the results were supported by the paper written by Mahler et al. on an interoception-based program on emotion regulation with BRIEF-2 [29]. When it came to emotional control, the interoception curriculum worked effectively [29].

The results presented in Table 6 show the results of the statistical analysis of the control and experimental groups' post-scores on the BRIEF-2 scale. Given that the p-value was <0.001 (significant at 1%), the alternative hypothesis was accepted. Consequently, there was a statistically significant difference in the post-test results for the behavior regimens in the experimental and control groups. This demonstrated that the experimental group's intervention showed more improvement than that of the control group. Analogous to this study, another study looked at interoception as a proactive strategy to reduce difficult behavior; the findings suggested the same conclusions as those provided by Goodall E [30]. According to the article, compared to those who did not utilize interoception, individuals who employed interoceptive activities reported significantly higher levels of engagement and learning in those groups [30].

We recognized that the small sample size and homogeneous sample contributed to the confined generalizability of our findings. The two components of academic self-regulation and behavioral problems have played a major role in children with learning disabilities. Future studies should also employ the correlation of two components, which are academic self-regulation and behavioral problems. It was often recognized that behavioral issues and self-regulation developed differently in different people. Large samples would also provide the opportunity to collect multiple data points for each construct to employ more complex statistical approaches and enable a more accurate evaluation between the two components. To confirm and expand on our findings, future studies should use larger sample sizes and a wider range of samples.

## Conclusions

Students with a learning disability are more likely to demonstrate poorer self-regulatory skills which may be a consequence of internalizing a history of repeated failures, frustrations, poor social interactions, and lower level of performance. Children can only grow and regulate themselves positively if they are able to know, which is what interoception makes possible. This study addressed all the aims and objectives and concluded that interoceptive activities appear beneficial for improving academic self-regulation and reducing behavioral problems among children with learning disabilities. although the vast majority of the study results point to positive impacts on health and therapy, further clinical trials are needed to assess interoception and self-regulation-based activities as a therapeutic strategy.
